# Palpation of the Lymphatic Ganglions Accessible to the Touch as a Clinical Diagnostic of Tuberculosis

**Published:** 1896-08

**Authors:** Paul Godbrille

**Affiliations:** Sanitary Inspector


PALPATION OF THE LYMPHATIC GANGLIONS ACCESSIBLE TO THE TOUCH AS A CLINICAL DIAGNOSTIC OF TUBERCULOSIS. By Mr. Paul Godbrille, Sanitary Inspector. If the tuberculin-test constitutes a valuable means of establishing the diagnosis of tuberculosis it should not be regarded as the only means, and other clinical tests which are at our disposal ought not to be wholly laid aside. Tuberculin is sometimes found to fail in the case of animals in which the disease is far advanced, and, on the other hand, there are circumstances where the veterinarian finds it difficult to follow up himself the rise in temperature; for example, the distance from his patient or the press of business which leaves him very little time. The examination of cattle in the markets no longer permits of the tuberculin-test. In these cases the practitioner must have recourse to all the means of investigation at his disposal, and very often by an analysis deduced from the symptoms he will succeed, by a process of elimination, in establishing a probable diagnosis. Ganglionic infection by its external manifestations often affords us aid, and ganglionic hypertrophies are of diagnostic import. Even in commencing hypertrophy these ganglions, concealed in the midst of the organs which lie near to them, reveal to the touch their pathological condition. It is for the purpose of indicating the mode of examining the different ganglions accessible to the touch and of judging of their condition that Mr. Godbrille has published the result of his investigations made during five years as cattle inspector in the market of La Villette. Normally the lymphatic ganglions are of elastic consistency and scarcely perceptible; under the influence of tuberculous infection they become enlarged, nodulated, and indurated; they are of woody consistency, and become soft in the centre, sometimes undergoing purulent disaggregation. The superior cervical ganglions and the guttural parotidean are most often infected. (1) The *submaxillary* ganglion situated at the edge of the bone on a level with the entrance of the sterno-maxillary muscle between the tendon of this latter and the maxillary gland has the volume of a dried almond covered with the woody rind. (2) The *protidean* ganglion, flattened into a strip running obliquely along the posterior surface of the neck of the condyle of the maxillary. It is covered by the parotid gland, but its lower extremity stretches out a little beyond it. (3) The *retro-pharyngean* ganglion, as large as the submaxillary, is situated beneath the "apophysis" across the atlas. (4) The superpharyngean or subsphenoidal ganglion, of the size of a dried almond not decorticated, supported by that of the opposite side, as if it were hung between the awl-shaped apophyses of the temporals beneath the mammillated eminences resulting from the welding of the basilary apophysis of the occipital with the sphenoidal. The examination of the maxillary ganglions can be made in a very simple way, but one must guard against confounding the hypertrophy of the same with goitre, which consists of an oblong tumor lying against the larynx. The parotidean ganglions can be felt by squeezing between the thumb and the other fingers the subcutaneous mass situated at the base of the concha and formed by the parotid and the annexed ganglion. The examination of the retro-pharyngean ganglions is important because these organs are often the seat of tuberculous indurations; they may even acquire the size of an apple. In order to feel them it is necessary to thrust the four fingers of each hand along each side of the throat at the same time, until the extremities of the medius are felt beneath the atloidian vault; one can then easily feel the ganglions. The supra-pharyngean ganglions cannot be well examined, but the hypertrophy of them leads often to respiratory trouble. The external ganglions of the trunk are very rarely the seat of tuberculous indurations, but nevertheless they ought not to be neglected, for the disease can show itself there. The prescapulary ganglion, from seven to ten centimetres long, situated at the bottom of the prescapulary groove (channel), and separated from the skin by the mastoideo-humeral, is easily displaced and felt by the four fingers when they are thrust beneath the point of the shoulder. · The axillary ganglions, three on each side, having the size of a kidney bean, are situated in the middle of the vein (veine) or avant-cœur (front heart) under the deep pectoral muscle. When they are affected with hypertrophy they can be felt in the middle of the chest. The prepectoral ganglions, which are so often affected, are unhappily not accessible to the touch. The precrural ganglion is a lymphatic cord, eight to ten centimetres in length, placed a little above the stifle-joint, and parallel to the anterior edge of the fascia lata, from which it is separated by three to four centimetres. The hollow of the flanks contains also three little ganglions which can be reached by way of the neoplasian processes and which have then the aspect of a dented ball. The mammary ganglions, which are very large, can be felt by seizing between the thumb and the other four fingers the two suspensory ligaments of the posterior quarters of the udder on a level with the entrance of the ischion. The sub-lumbar ganglions even can be examined, in case of necessity, through the rectum.—*Recueil de Médecine Vétérinaire*, 15 Sept., 1895. In the *Annales de Méd. Vét.*, Jan. and Feb., 1896.

